# Molecular and behavioural abnormalities in the FUS‐tg mice mimic frontotemporal lobar degeneration: Effects of old and new anti‐inflammatory therapies

**DOI:** 10.1111/jcmm.15628

**Published:** 2020-07-15

**Authors:** Johannes de Munter, Diana Babaevskaya, Erik Ch. Wolters, Dmitrii Pavlov, Ekaterina Lysikova, Allan V. Kalueff, Anna Gorlova, Margarita Oplatchikova, Igor A. Pomytkin, Andrey Proshin, Aleksei Umriukhin, Klaus‐Peter Lesch, Tatyana Strekalova

**Affiliations:** ^1^ Department of Psychiatry and Neuropsychology School for Mental Health and Neuroscience Maastricht University Maastricht The Netherlands; ^2^ Laboratory of Psychiatric Neurobiology, Institute of Molecular Medicine and Department of Normal Physiology I.M. Sechenov First Moscow State Medical University Moscow Russia; ^3^ Laboratory of Cognitive Dysfunctions Institute of General Pathology and Pathophysiology Moscow Russia; ^4^ Department of Advanced Cell Technologies, Institute of Regenerative Medicine I.M. Sechenov First Moscow State Medical University Moscow Russia; ^5^ Institute of Physiologically Active Compounds Moscow Russia; ^6^ Faculty of Biology Ural Federal University Ekaterinburg Russia; ^7^ Institute of Translational Biomedicine and Institute of Experimental Medicine St. Petersburg State University and Almazov Medical Research Center St. Petersburg Russia; ^8^ Department of Advanced Cell Technologies I.M. Sechenov First Moscow State Medical University Moscow Russia; ^9^ PK Anokhin Research Institute of Normal Physiology Moscow Russia; ^10^ Division of Molecular Psychiatry Center of Mental Health University of Würzburg Würzburg Germany

**Keywords:** amyotrophic lateral sclerosis, animal model, celecoxib, emotionality and cognition, frontotemporal lobar degeneration, FUS[1‐359]‐tg mice, neuroinflammation, riluzole, stem cell therapy

## Abstract

Genetic mutations in FUS, a DNA/RNA‐binding protein, are associated with inherited forms of frontotemporal lobar degeneration (FTLD) and amyotrophic lateral sclerosis (ALS). A novel transgenic FUS[1‐359]‐tg mouse line recapitulates core hallmarks of human ALS in the spinal cord, including neuroinflammation and neurodegeneration, ensuing muscle atrophy and paralysis, as well as brain pathomorphological signs of FTLD. However, a question whether FUS[1‐359]‐tg mouse displays behavioural and brain pro‐inflammatory changes characteristic for the FTLD syndrome was not addressed. Here, we studied emotional, social and cognitive behaviours, brain markers of inflammation and plasticity of pre‐symptomatic FUS[1‐359]‐tg male mice, a potential FTLD model. These animals displayed aberrant behaviours and altered brain expression of inflammatory markers and related pathways that are reminiscent to the FTLD‐like syndrome. FTLD‐related behavioural and molecular Journal of Cellular and Molecular Medicine features were studied in the pre‐symptomatic FUS[1‐359]‐tg mice that received standard or new ALS treatments, which have been reported to counteract the ALS‐like syndrome in the mutants. We used anti‐ALS drug riluzole (8 mg/kg/d), or anti‐inflammatory drug, a selective blocker of cyclooxygenase‐2 (celecoxib, 30 mg/kg/d) for 3 weeks, or a single intracerebroventricular (i.c.v.) infusion of human stem cells (Neuro‐Cells, 500 000‐CD34^+^), which showed anti‐inflammatory properties. Signs of elevated anxiety, depressive‐like behaviour, cognitive deficits and abnormal social behaviour were less marked in FUS‐tg–treated animals. Applied treatments have normalized protein expression of interleukin‐1β (IL‐1β) in the prefrontal cortex and the hippocampus, and of Iba‐1 and GSK‐3β in the hippocampus. Thus, the pre‐symptomatic FUS[1‐359]‐tg mice demonstrate FTLD‐like abnormalities that are attenuated by standard and new ALS treatments, including Neuro‐Cell preparation.

## 1 INTRODUCTION

Genetic mutations in FUS, which is DNA/RNA‐binding protein, can cause frontotemporal lobar degeneration (FTLD) and amyotrophic lateral sclerosis (ALS).^1^ FTLD is a debilitating disease, often accompanying ALS and involving atrophy of the frontal/temporal lobes and affecting emotional, social and cognitive functions.^2^ Until recently, the contribution of FUS to the FTLD/ALS pathology remains poorly understood. The available FTLD models based on the FUS mutation often report somewhat non‐specific behavioural deficits and limited brain pathology with a late onset,^3^ or the very rapid development of physiological and motor ALS‐like pathology^4^, and, for this reason, it has been argued that further refinement is required.^5^ The construction of the FUS[1‐359]‐tg mice, expressing truncated human FUS[1‐359], has been shown to exhibit many of the hallmark characteristics of ALS,^6^ and FTDL‐like changes during the pre‐symptomatic stage.^7^ This model provides a promising tool to explore the temporal contribution of FUS mutations on molecular and behavioural outcome. Meanwhile, hitherto the FTDL‐like behavioural features and the accompanying molecular changes have not been investigated. Here were addressed this outstanding issue by studying the impact of therapy used in the clinic and new anti‐inflammatory therapies on emotional, cognitive and social abnormalities in FUS‐tg animals. These therapies have been shown to reduce the ALS‐like pathology in these mutants.^8^

## 2 METHODOLOGY

Animals, study design, methodology and statistical analysis are described in a Appendix S1 (see also Figure S1). At the age of eight weeks, which is considered to be the beginning of adulthood, the FUS[1‐359]‐tg (FUS‐tg) male mutants display no signs of neurodegeneration in the CNS (Figure S3), nor any motor deficits.^6^, ^8^ For this reason we selected mice of this age for both Study 1 and Study 2. FUS‐tg mice used here showed no deficits in motor tests (*not shown*). Over the course of 5 days, wild‐type (WT) and FUS‐tg mice of average age of 9 weeks were investigated for (a) time spent in the open arms of the O‐maze, (b) sucrose preference, (c) the duration of immobility in the tail suspension test, (d) number of rears in the novel cage test, (e) displacement of pellets in the marble test, and (f) the duration of attacks and tail rattling in the resident‐intruder test.^9^ The FTLD syndrome is well documented to be associated with pathological changes in the limbic system, including the hippocampus and prefrontal cortex and underlie emotional, social and cognitive abnormalities.^5^ These two structures play pivotal roles in the regulation of social, anxiety‐ and depressive‐like behaviours, as well as exploration and cognitive tasks in the mouse models employed here ^9^ and, hence, were studied for potential molecular changes (Figure S1A). Therefore, mice were killed and the hippocampus and the prefrontal cortex collected for RNA isolation/cDNA synthesis and RT‐PCR assay of FTDL‐related pro‐inflammatory markers: tumour necrosis factor (TNF), cyclooxygenase‐1 (COX‐1), interleukin‐1β (IL‐1β) and cytokine expression regulatory molecules implicated in the ALS pathology, matrix‐metalloproteinase‐9 (MMP‐9) and tissue inhibitor of metalloproteinase‐1 (TIMP‐1).^2^ For experimental details, see Appendix S1 and Table S1).

Then, *per os* administration of the commonly used ALS treatment riluzole (8 mg/kg/d), oranti‐inflammatorycyclooxygenase‐2 blocker celecoxib (30 mg/kg/d), or vehicle, to FUS‐tg and WT mice of average age of 9 weeks was started; or they received an intracerebroventricular (i.c.v.) infusion of Neuro‐Cells (1.39 × 10^6^ mesenchymal and haemopoietic human stem cellscontaining 5 × 10^5^ of CD34^+^ cells) or Ringer‐solution,^8^, ^10^ for details on Neuro‐Cells preparation, see Appendix S1 and Tables S3 and S4). Two weeks later, over a period of 5 days, mice were behaviourally scored for (a) time spent in lit box in the dark/light test, (b) new object recognition index, (c) the duration of immobility in the tail suspension test, and (d) duration of following, number of attacks and of tail rattling in the resident‐intruder test.^11^ A different battery of tests was employed in the second set of animals with the aim of providing an extended behavioural characterization of new FUS‐tg line (Figure S1B). To compare molecular changes of mutants in brains to the reported changes in the spinal cord, mice were killed at age of 15 weeks, and the hippocampus and the prefrontal cortex were dissected for Western blots for IL‐1β, Iba‐1, glycogen‐synthase‐kinase‐3 (GSK‐3)‐β and GSK‐3α.^8^, ^10^ For experimental details, see Appendix S1 and Table S2).

Data were treated by unpaired two‐tailed *t* test, or one‐ or two‐way ANOVA followed by Tukey's test, for two or multiple group comparisons, respectively. Repeated measures were analysed by two‐way ANOVA for repeated measures followed by Sidak post hoc test.

## 3 RESULTS AND DISCUSSION

For full statistical results, see Supplementary Tables.

### 3.1 Pre‐symptomatic FUS‐tg mice display aberrant behaviours and pro‐inflammatory changes

**FIGURE 1:**
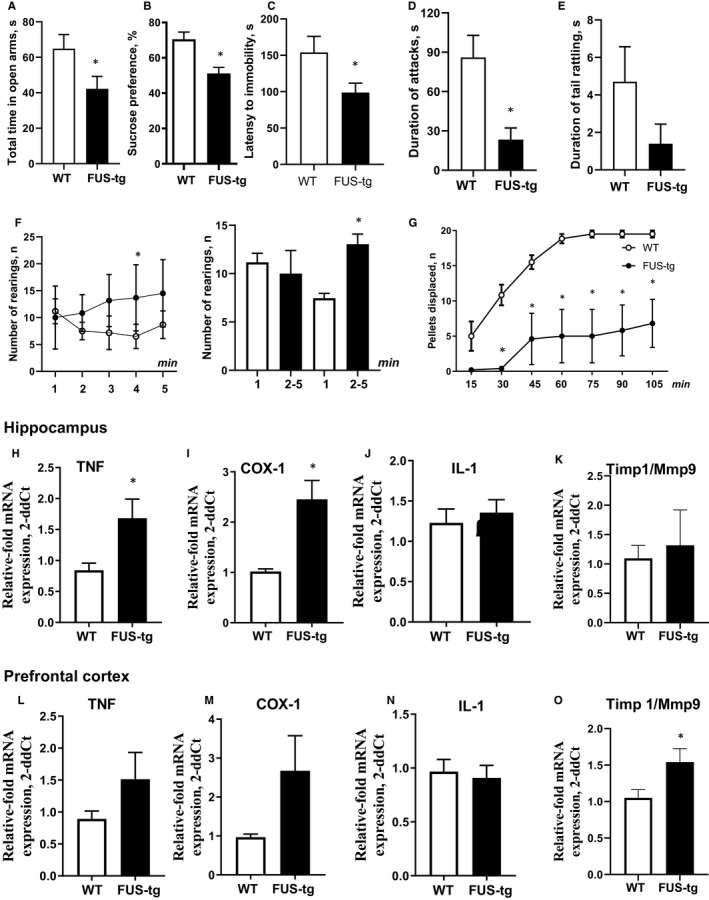
FTLD‐like changes in FUS‐tg mice in (A‐G) behavioural parameters and (H‐O) molecular markers; *P < .05: FUS‐tg (n = 9‐15) vs WT (n = 8‐14) groups, unpaired *t* test and repeated ANOVA and Tukey's test (see the text)

FUS‐tg mice spent less time in open arms of the elevated O‐maze and exhibited a reduced latency to immobility in the tail suspension test, decreased sucrose preference, reduced duration of attacks (*P* < .05 vs controls, unpaired *t* test; Figure 1A‐D), and the duration of tail rattling was unchanged (*P* > .5; Figure 1E; Table S5A). A lack of group differences in general locomotion in the O‐maze rules out a possibility of non‐specific confounds in behavioural analysis of two genotypes (Figure S3). Two‐way ANOVA for repeated measures revealed significant time x genotype interaction and genotype effect in the number of rears in the novel cage test (*P* < .05; Table S5B). Sidak post hoc test for multiple comparisons showed significant increase of this measure in FUS‐tg mice on the 4th min (*P* < .05 vs WT mice; Figure 1F) and trends to such changes on minutes 2, 3 and 5 (*P* > .05, Table S5B). In comparison with controls, mutants exhibited unchanged activity on the 1st min of scoring (*P* > .5, *t* test; Table S5C) and significantly higher number of rearings averaged over the 2nd‐5th min of the test (*P* < .05, *t* test), thus, showing disrupted novelty adaptation (Figure 1F).

There were significant time and genotype effects, as well as significant time x genotype interaction the in number of pellets displaced during the marble test (*P* < .05, two‐way ANOVA for repeated measures; Table S5D). FUS‐tg mice displaced fewer pellets during all time periods of the test, except for 0‐15 minutes, suggesting deficient hippocampus‐dependent performance (*P* < .05 vs WT mice, Sidak post hoc test; Figure 1G; Table S5D).

Signs of helplessness, anhedonia, anxiety‐like features, cognitive and social abnormalities in FUS‐tg animals are characteristic of other FTDL paradigms, that is progranulin‐deficient mice,^12^ ΔNLS‐FUS mice^3^ and TDP‐43‐tg mice.^13^ The current study suggests the most prominent FDTL‐related deficits of the FUS‐tg mice are the classic tests of emotionality, social and hippocampus‐dependent performance. In comparison with other FTLD rodent models based on the FUS mutation, the findings reported here in the FUS‐tg mice suggest that no hyperactivity or other non‐specific behavioural alternations are present, but, typical for this disease, features of anxiety, apathy, cognitive and social deficits are observed.^3^, ^5^, ^7^ Hippocampal concentrations of TNF and COX‐1 mRNA were elevated in the FUS‐tg mice (*P* < .05 vs controls; unpaired *t* test; Figure 1H‐M, Table S6). The Timp1/Mmp9 ratio was augmented in the prefrontal cortex (*P* < .05, *t* test; Figure 1J‐K), and no other group differences were present (*P* > .5, *t* test). Together, these data provide evidence of pro‐inflammatory changes within the brain of the FUS‐tg mice that a characteristic for FTDL‐syndrome^14^ and aberrant Timp1/Mmp9 ratio, a factor of ALS pathology,^2^ cytokine expression^15^ and neuronalplasticity.^16^, ^17^

### 3.2 Ameliorative effects of standard ALS treatments and ‘Neuro‐cells’ on the behavioural and molecular changes in the FUS‐tg mice

**FIGURE 2:**
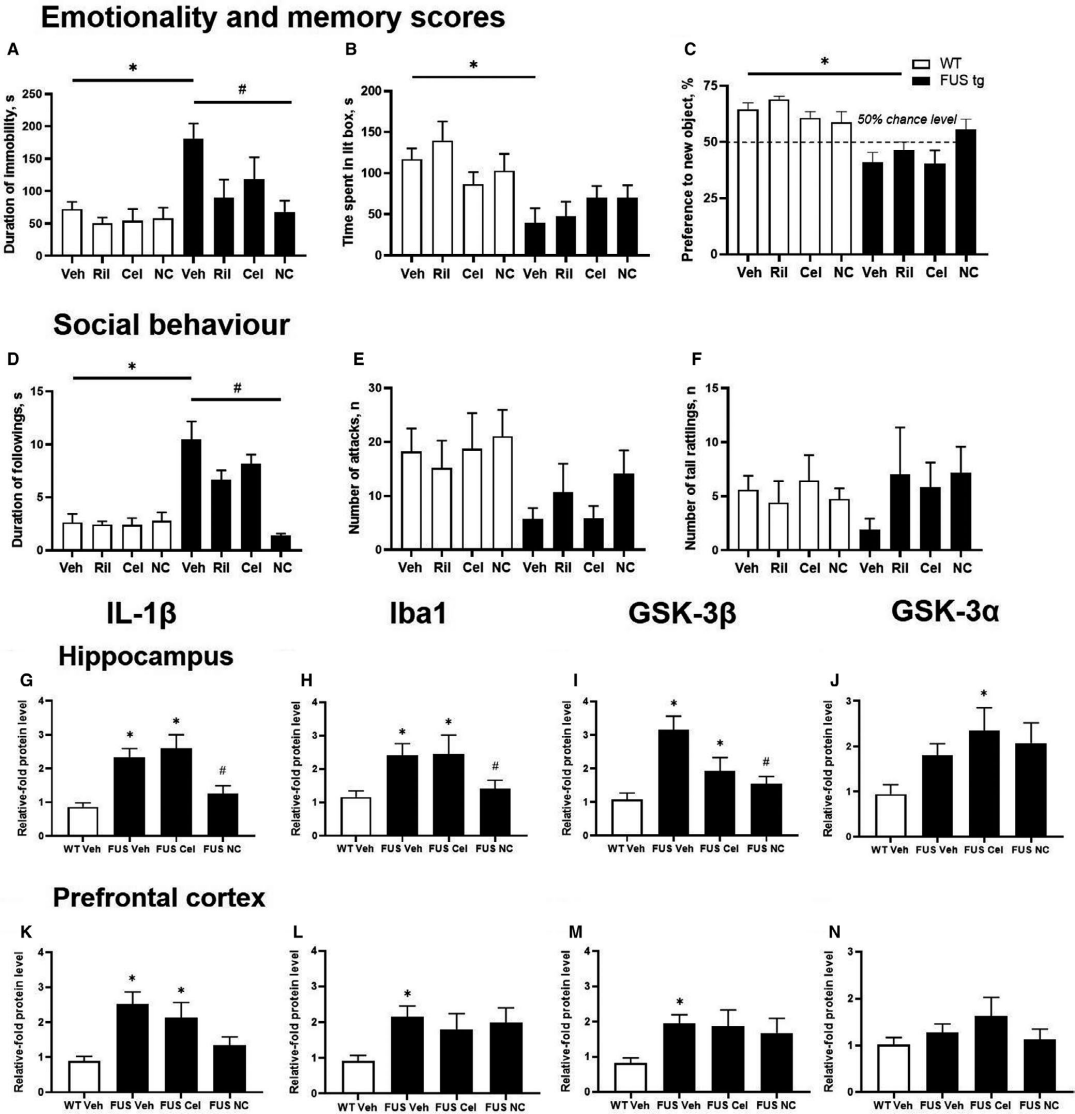
Ameliorative effects of standard ALS treatments and Neuro‐Cells in FUS‐tg mice on (A‐F) behaviours and (G‐N) expression of FTLD/ALS‐related molecular markers;*P < .05, vs respective WT‐group (n = 6‐12), #*P* < .05, vs FUS‐tg‐Veh group (n = 7‐14); ANOVA and Tukey's test (see the text)

Concerning the duration of immobility, there were no significant effects of genotype, treatment, or their interaction (*P* > .5, two‐way ANOVA, Table S7), but FUS‐tg‐Veh displayed elevated immobility scores (*P* < .05, vs WT‐Veh group, Tukey's test; Figure 2A), that were not observed in the treated mutants (*P* > .05, Tukey's test). In comparison with FUS‐tg‐Veh mice, this behaviour was decreased in FUS‐tg‐NC, but not FUS‐tg‐Ril and FUS‐tg‐Cel animals (*P* < .05 and *P* > .5, respectively; Tukey's test). For the time spent in the lit box, there were no significant effects of treatment, a genotype x treatment interaction (*P* > .5, two‐way ANOVA), but there was an impact of genotype (*P* < .05, two‐way ANOVA). In comparison with respective wild‐type controls, FUS‐tg‐Veh animals, but not treated mutants exhibited a shortened duration of time spent in the lit box (*P* < .05 and *P* > .5, respectively, Tukey's test; Figure 2B, Table S7), which was increased in FUS‐tg‐NC vs FUS‐tg‐Veh animals (*P* < .05, Tukey's test). No other group differences were found.

There were no group differences in the object exploration (*P* > .5, two‐way ANOVA and Tukey's test; *not shown*). A significant genotype x treatment interaction and effects of genotype (*P* < .05, two‐way ANOVA), not of treatment (*P* > .5, two‐way ANOVA), were found for the preference for the new object. All mutants except FUS‐tg‐NC animals had decreased scores for this measure (*P* < .05 and *P* > .05, vs WT groups, respectively, Tukey's test; Figure 2D, Table S7). A significant genotype x treatment interaction was revealed for the duration of ‘following’ (*P* < .05 and *P* > .5, respectively, two‐way ANOVA), but not for numbers of attacks and tail rattling (*P* > .5, Tukey's test; Figure 2E,F, Table S7). There was a significant genotype effect on the duration of following and number of attacks, but no significant treatment group differences (*P* > .05, two‐way ANOVA). We found a significant treatment effect on the duration of following (*P* < .05, two‐way ANOVA), which was increased in FUS‐tg‐Veh and FUS‐tg‐Cel mice (*P* < .05, Tukey's test vs respective control groups; Figure 2D, Table S7), and FUS‐tg‐Cel group showed its decrease vs FUS‐tg‐Veh group (*P* < .05, Tukey's test). No other group differences were found. Thus, treatments ameliorated the behavioural end‐points in the FUS‐tg mice, where their effects on the measures of anxiety and aggression were particularly profound, suggesting their importance in the evaluation of FDTL‐like features in this mouse line.

Hippocampal concentrations of IL‐1β, Iba‐1, GSK‐3β and GSK‐3α were different between the groups (*P* < .05; one‐way ANOVA, Figure 2G‐J, Figure S4A; Table S8A). FUS‐tg‐Veh animals exhibited increased expression of all molecules except GSK‐3α (*P* < .05 and *P* > .05, vs WT‐Veh; respectively; Tukey's test). In FUS‐tg‐NC mice, concentrations of IL‐1β and GSK‐3βwere lower than in the FUS‐tg‐Veh group (*P* < .05, Tukey's test). In the prefrontal cortex, levels of IL‐1β, Iba‐1 and GSK‐3β were different between the groups (*P* < .05), but not for GSK‐3α (*P* > .05 and *P* > .05, respectively, Tukey's test). FUS‐tg‐Veh animals displayed elevated expression of the target molecules (*P* < .05, vs WT‐Veh), except GSK‐3α (*P* > .05, Tukey's test; Figure 2K‐N, Figure S4B, Table S8B). Concentrations of IL‐1βwere found to be decreased in FUS‐tg‐NC vs FUS‐tg‐Veh mice (*P* < .05, Tukey's test). No other group differences were found. In summary, FUS‐tg mutants displayed up‐regulated protein expression for the inflammatory markers and for GSK‐3, which was reminiscent of the changes observed in the spinal cord^4^ and were sensitive to the treatments. Thus, behavioural and molecular abnormalities of FUS‐tg mice were overly reduced by the use of Rilusole, celecoxib or Neuro‐Cells, which often exerted greater effects.

## 4 CONCLUSIONS

Together, the pre‐symptomatic FUS[1‐359]‐tg mice demonstrate behavioural changes that are reminiscent of the FTLD‐syndrome abnormalities, and they are attenuated by all the treatments. Hence, FUS[1‐359]‐tg mutant mice can be exploited as a new paradigm of the FTLD to address molecular mechanisms underlying this disease and test new treatment options.

## CONFLICT OF INTEREST

There is no conflict of interest.

## AUTHOR CONTRIBUTIONS

**Johannes de Munter:** Funding acquisition (lead); investigation (lead); methodology (supporting); resources (supporting); writing‐review and editing (supporting). **Diana Babaevskaya:** Investigation (equal); methodology (equal); visualization (equal); writing‐original draft (equal). **Erik Wolters:** Conceptualization (supporting); data curation (supporting); funding acquisition (supporting); resources (supporting); writing‐review and editing (supporting). **Dmitrii Pavlov:** Formal analysis (equal); investigation (equal); visualization (supporting); writing‐original draft (supporting). **Ekaterina Lysikova:** Investigation (supporting); methodology (supporting); project administration (equal); writing‐review and editing (supporting). **Allan Kalueff:** Conceptualization (equal); formal analysis (supporting); writing‐review and editing (supporting). **Anna Gorlova:** Formal analysis (supporting); methodology (supporting); visualization (supporting); writing‐review and editing (supporting). **Margarita Oplatchikova:** Investigation (supporting); methodology (supporting). **Igor A. Pomytkin:** Conceptualization (supporting); data curation (equal); formal analysis (supporting); methodology (supporting); validation (equal); writing‐original draft (supporting). **Andrey Proshin:** Formal analysis (supporting); investigation (supporting); project administration (supporting); supervision (supporting); writing‐review and editing (supporting). **Aleksei Umriukhin:** Conceptualization (supporting); formal analysis (supporting); project administration (supporting); resources (equal); supervision (equal); writing‐review and editing (supporting). **Klaus‐Peter Lesch:** Conceptualization (equal); data curation (equal); formal analysis (equal); funding acquisition (equal); resources (equal); writing‐review and editing (supporting). **Tatyana Strekalova:** Conceptualization (lead); funding acquisition (supporting); methodology (lead); project administration (lead); resources (lead); supervision (lead); writing‐original draft (equal).

## Supporting information

Supplementary Material

## Data Availability

Experimental details of this work are available on request.
