# Supplementary material for: Molecular and behavioural abnormalities in the FUS‐tg mice mimic frontotemporal lobar degeneration: Effects of old and new anti‐inflammatory therapies
Source: J Cell Mol Med. 2020 Jul 15;24(17):10251–7. doi: 10.1111/jcmm.15628 (PMC7520339; doi:10.1111/jcmm.15628)
Supplement: Supplementary file 1 — Supplementary Material [file JCMM-24-10251-s001.docx]

**Supporting File**

**MATERIALS AND METHODS**

**Animals and housing conditions**

A colony of FUS-transgenic mice (FUS-tg) and their wild type littermates (WT) was bred and housed in the FDA-certified SPF facilities of the IPAC Center of Pre-clinical Trials ([https://www.google.de/search?client=firefox-b&dcr=0&biw=1280&bih =589&tbm=isch&sa=1&ei= 2Ud_WvCPK4PvgAaAxq2Aag & q=Institute+ of+Physiologically+ Active+Compounds%2C +Russian+Academy+of+Sciences%2C +Chernogolovka%2C+Moscow+region&oq=Institute+of+Physiologically+Active+ Compounds%2C+Russian+ Academy+ of+Sciences%2C+ Chernogolovka%2C+Moscow+ region&gs_l=psy-ab.3.. 13217.16388.0.16904. 45.10.0.0.0.0.0.0..0.0....0...1c.1.64.psy-ab..45.0.0....0.ISJYlZSeqkU#imgrc=9D26kkQ71OzfeM](https://www.google.de/search?client=firefox-b&dcr=0&biw=1280&bih%20=589&tbm=isch&sa=1&ei=%202Ud_WvCPK4PvgAaAxq2Aag%20&%20q=Institute+%20of+Physiologically+%20Active+Compounds%2C%20+Russian+Academy+of+Sciences%2C%20+Chernogolovka%2C+Moscow+region&oq=Institute+of+Physiologically+Active+%20Compounds%2C+Russian+%20%20Academy+%20of+Sciences%2C+%20Chernogolovka%2C+Moscow+%20region&gs_l=psy-ab.3..%2013217.16388.0.16904.%2045.10.0.0.0.0.0.0..0.0....0...1c.1.64.psy-ab..45.0.0....0.ISJYlZSeqkU#imgrc=9D26kkQ71OzfeM)). Male mice of both genotypes, 9-10-weeks-old at the start of the experiment (average age of 9-weeks), were single housed in standard plastic cages (27x22x15) and maintained on a 12-hour light/dark cycle (lights on at 21:00), under controllable laboratory conditions (22±1°C, 55% humidity, room temperature 22-24ºC), food and water were available ad libitum. Housing conditions and all experimental procedures were set up in accordance with a Directive 2010/63/EU of 22 September 2010 and carried out under approval of the Committee for Bioethics (N19-16.06.2017) of IPAC RAS. Bone marrow collection from healthy volunteers was carried out under GMP license (Neuroplast BV Farmatec The Netherlands) and approved by Ethical Committee of MUMC, Maastricht University (iCell1 METC MUMC and iCell2 METC Zuyderland Zuid). All efforts were undertaken to minimize the potential discomfort of experimental animals.

**Production of FUS Transgenic Mice**

A generation of FUS-tg mice was performed as describe elsewhere (Schelkovnikova et al., 2013). Briefly, a fragment of human FUS[1–359] cDNA including 9 bp of 5′-UTR was cloned into Thy-1 promoter plasmid 323-pTSC21k. A gel-purified fragment obtained by digestion of the resulting plasmid DNA with NotI was used for microinjection of mouse oocytes. Transgenic animals were identified by PCR analysis of DNA from ear biopsies by the presence of 255-bp product (primers 5′-TCTTTGTGCAAGGCCTGGGT-3′ and 5′-AGAAGCAAGACCTCTGCAGAG-3′). Initially produced transgenic line on C57Bl6/CBA genetic background was backcrossed with CD1 wild type mice by seven or more generations.

**Study design**

In *Study 1*, FUS-tg mice at the age of 9 weeks corresponding to a pre-symptomatic stage of the ALS model along with wild type littermates, were studied for motor functions in the Cat-walk, Wire test, Grip-test, Pole test and Rotarod model with 1-hour inter-test interval as described elsewhere (Veniaminova et al., 2020; de Munter et al., 2020; *see below*) to verify the absence of any motor deficits during three days prior the experiment (Fig.1A, *data now shown*). These mice were investigated in the tests for emotionality, social behaviour and hippocampus-dependent performance thereafter. On Day 1, mice were studied in the elevated O-maze test for anxiety-like behaviour, on Day 2, they were studied in the sucrose preference test for a sensitivity to reward, on Day 3, mice were scored for novelty exploration in the novel cage test and despair behaviour in the tail suspension test, on Day 4, mice were investigated for hippocampus-dependent performance of food pellet displacement behaviour in the marble test and on Day 5, parameters of social interaction were scored in the resident-intruder test (*see below*). Seventy two hours after the last test mutant animals were were perfused with NaCl, anaesthetized and killed, alone with respective wild type controls (*see below*) and brains were harvested and dissected to the hippocampus and the prefrontal cortex for a subsequent molecular analysis with RT PCR (*see below*). Twelve animals in average were used per group; numbers of animals used is indicated in Figure legends.

In *Study 2*, FUS-tg mice at the same age as in the *Study 1* were tested for motor functions as described above; no motor deficits were found in mutants (*data not shown*). FUS-tg and WT mice were treated with (**1**) regular tap water, or (**2**) riluzole (8 mg/kg/day, via drinking water), or (**3**) or celecoxib (30mg/kg/day) via diet pellets, or (**4**) single stereotaxic i.c.v. infusion of Neuro-Cells (NC, 500,000-CD34^+^ in 10 ul of Ringer Lactate buffer), or (**5**) stereotaxic i.c.v. infusion of Ringer Lactate buffer (Fig.1B; de Munter et al., 2020). Doses of pharmaca and stereotaxic surgery were carried out as described elsewhere (de Munter et al., 2020; *see below*). Ten animals in average were used per group; numbers of animals used is indicated in Figure legends.

During the following two weeks, all mice were weekly weighed and investigated in the rotorod, pole and wire tests for motor functions, dosing with drugs was continued. As post-surgery physiological parameters of wild type mice that received either tap water or i.c.v. vehicle injection were similar (*data not shown*), these two groups were merged into a vehicle-treated group for subsequent group comparisons. On the third week, mice were investigated for emotional, social and cognitive behaviors. For the sake of a more complete characterization of this new line of FUS-tg mice, we have chosen to employ a battery of behavioural models similar to Study 1, although with an extended scope of behavioral responses, while providing a sufficient consistency in the analysis of behaviour between the two studies. At this point of study, no signs of motor deficits in were found in FUS-tg groups (*data not shown*).

On Day 1, mice were scored for anxiety-like behavior in the dark/light test, on Days 2 and 3, mice new object recognition index was assessed, on Day 4, mice were studied in the tail suspension test, and on Day 5, the resident-intruder test was performed (*see below*). To verify whether brain molecular changes in mutants match those previously reported for spinal cord in symptomatic FUS-tg animals, mice were killed at their symptomatic age of fifteen weeks, given in average age of the onset of the ALS-like syndrome of 14 weeks (Shelkovnikova et al., 2013; deMunter et al., 2020). Dosing of pharmaca was continued for the remaining three weeks and was interrupted 48 h prior a sacrifice. Mice were anaesthetized, perfused with NaCl and killed, brains were isolated, the hippocampus and the prefrontal cortex were dissected as described above, for Western blot assay. Brain protein expression of target proteins was measured by Western blot assay in mice treated with either vehicle, Neuro-Cells or celecoxib, since distinct expression changes in the lumbar parts of spinal cord previously showed in these groups for selected markers of inflammation and cellular distress (de Munter et al., 2020). Pharmaca outline and genotype were double blind for all experimenters.

**Supplementary Figure 1. Experiment design of study on** **(A)** naïve FUS-tg mice and **(B)** FUS-tg mice treated with standard ALS treatments and “Neuro-Cells”.

**Behavioural testing**

The person performing the experiments was blind for the genotype and treatment until the end of the behavioral tests. Experiments were carried out in the same rooms by the same persons between 09:00 and 17:00 h. Animals were allowed to adapt to experimental room for at least 1 h before testing. All experiments were recorded on videotape and scored manually and by the means of ViewPoint software (ViewPoint SA, Lissieu, France).

*Rotorod*

Mice were placed on constantly rotting rod of rotarod (Columbus Instruments, Columbus, OH, USA; speed 10 rpm) for 600s. Latency to fall and the number and percent of mice with falling events (latency<200s) were registered in three runs as described elsewhere (Veniaminova et al., 2020; de Munter et al., 2020).

*Wire test*

Mice were allowed to grip a horizontal wire (diameter 0.3 cm, height above the surface 60 cm) for 180s. The latency of falling and the number and percent of mice with falling events (latency<20s) were recorded as described elsewhere (Veniaminova et al., 2020; de Munter et al., 2020).

*Pole test*

Mice were placed on a top of the vertical bar (diameter 1.1 cm, height 60 cm) and allowed to climb down to a horizontal surface. The latency of descending the bar and the number and percent of mice with sliding events (latency to descend<50s) were scored as described elsewhere (Veniaminova et al., 2020; de Munter et al., 2020).

*Grip test*

The grip strength meter (Bioseb, Vitrolles, France) was positioned horizontally and mice were held by the tail and lowered towards the apparatus. The mouse was allowed to grab the metal grid bar and are then pulled backwards in the horizontal plane with minimal force sufficient to make it losing a grip. The measurement was accomplished using a sensor and was expressed in units as described elsewhere (de Munter et al., 2020).

*CatWalk test*

CatWalk XT (Noldus, Wageningen, Netherlands) was employed for refined quantitative assessment of motor functions in experimental groups of mice. In the current study, the duration of right and left hindlimb swing was measured in baseline conditions and after the onset of the treatment as described elsewhere (de Munter et al., 2020). These measures were defined as the most sensitive and reliable parameters of motor functions in the FUS-tg mice in pilot experiments (Lysikova and Ninkina, *unpublished results*).

*O-maze test*

The apparatus (Open Science, Moscow, Russia), which consisted of a circular path (runway width 5.5cm, diameter 46cm), was placed 50cm above the floor. Two opposing arms were protected by walls (height 10cm), and the illumination strength was 25 lx. Anxiety-like behavior was assessed using previously validated parameters as described elsewhere (Couch et al., 2016; Costa-Nunes et al., 2020). Mice were placed in one of the closed arm compartments of the maze. Total duration of time spent in the open arms of the maze was scored as the parameter of anxiety-like behaviour during 5 min. Number of crossed sections (4x5.5cm) in both arms was scored as well.

*Sucrose Preference*

Mice were given 8 hours of free choice between two bottles of either 1% sucrose or standard drinking water, as described elsewhere (Strekalova et al., 2004, Couch et al., 2013; Pavlov et al., 2019). Bottles were weighed before and after conducting the sucrose preference, and consumption calculated accordingly. The beginning of the test started with the onset of the dark (active) phase of animals’ cycle. To prevent the possible effects of side preference in drinking behaviour, the position of the bottles in the cage was switched at 4 hours, halfway through testing. No previous food or water deprivation was applied before the test. Other conditions of the test were applied as described elsewhere (Strekalova and Steinbusch 2010). Percentage preference for sucrose is calculated using the following formula: Sucrose Preference = Volume (Sucrose solution)/(Volume (Sucrose solution) + Volume (Water) x 100.

*Novel Cage Test*

The 5-min long novel cage test was carried out to assess exploration of a new environment as described elsewhere (Strekalova et al., 2004, Couch et al., 2016; Veniaminova et al., 2020). Mice were introduced into a standard plastic cage (21 cm x 21 cm x 15 cm) filled with fresh sawdust. The number of exploratory rears was counted under red light per each minute, and summed up for minutes 2-5 of the test.

*Tail suspension test*

Mice were subjected to the tail suspension by being hung by their tails with adhesive tape to a rod 50 cm above the floor for 6 min. Animals were tested in a dark room where only the area of the modified tail suspension construction was illuminated by a spotlight from the ceiling; the lighting intensity on the height of the mouse position was 25 Lux. The total duration of immobility was scored according to the protocol that was previously validated with Noldus EthoVision XT 8.5 (Noldus Information Technology, Wageningen, Netherlands) as described elsewhere (Malatynska et al., 2012). In accordance with the commonly accepted criteria of immobility, the immobility behaviour was defined as the absence of any movements of the animals' head and body.

*Pellet displacement marble test*

All experimental groups were tested for pellet displacement in a marble test as described elsewhere (Strekalova et al., 2013; Veniaminova et al., 2017, 2020). A tendency to displace small objects, e.g. small stones or food pellets, from a tube inside the cage, is species-specific in mice and has been demonstrated to depend on an intact hippocampal formation. Using a paper tube (internal diameter 4 cm, length 10 cm), filled with 20 food pellets and placed in the middle of a home cage (21 cm × 27 cm × 14 cm), the number of food pellet displaced by each mouse was assessed every 15 min during 1 h and 45 min.

*Resident-intruder test*

In the resident-intruder test, mice were individually placed in an observation cage (30x60x30 cm) for 30 min. Then, each mouse was exposed to a previously group-housed naïve C57BL/6N male intruder of similar weight and age for 8 min in the same cage. The duration and the number of attack and tail rattling, and the duration of following behavior were scored as described elsewhere (Couch et al., 2016; Strekalova et al., 2018; Gorlova et al., 2020; Veniaminova et al., 2020).

*Dark/Light Box Test*

Mice were placed into the black compartment (15х20х25 cm) from where they could visit the lit box (30х20х25 cm, Open Science, Moscow, Russia). Time spent by mice in the lit part of the box, in which illumination intensity was 25 Lux on the surface of the apparatus, was scored during 5-min period, as described elsewhere (Costa-Nunes et al., 2013; Strekalova et al., 2018).

*Object Recognition Memory test*

Mice were placed in a glass cylinder observation chamber (Ø 25 cm, height 35 cm) that was situated on a stand of 1 m high placed by two walls of the lab room. The cylinder was 5cm from the edge on two opposite sides and 30 cm from the edge on the other two sides. Using sound-proof conditions with subtle illumination (5 Lux) animals were allowed to explore two identical objects (taste-free and smell-free plastic toys of 6 cm x 3 cm x 2.5 cm) which were located in either part of the cylinder or 15 min. Previous experiments (Strekalova et al., 2013) have shown that object placed by the edge is explored by C57Bl6 mice in average 50% less than the same object at the opposite location that is likely to be owing to their species-specific tegmotaxic traits and fear of height.

On the Day 1, animals were allowed to explore two objects placed either close to the walls “preferable” zone (the area in 30 cm from edge) or distanced from walls “non-preferable” zone (the area in 5 cm from edge) in the observation cylinder. On the Day 2, the test was repeated. The object from “non-preferred” zone was replaced with a new object of a similar size (3 cm x 6 cm x 3 cm) and of exactly the same material. A placement of a new object to “non-preferred” area meant to contrast behavioral manifestation of novelty exploration under competing motivations of animals of staying by the walls and avoid the height. The duration of exploration of each object was recorded on each day. Preference for the object exploration was calculated as a percentage of the duration of exploration of a new object from the total duration of exploration of both objects on Day 2: Index of object preference (recognition) = [Object exploration /Total exploration of two objects] × 100%. An increased preference in the exploration of a new object that has replaced the former one in the “non-preferred” area from day 1 to day 2, was taken as an index of recognition (memory) of the former object.

**Killing and Tissue Collection**

Mice were terminally anaesthetized with an intraperitoneal injection of sodium pentobarbitone. The left ventricle was perfused in situ with 10 mL ice-cold saline; the brain of each mouse was dissected and the prefrontal cortex and the hippocampi as described elsewhere (Trofimov et al., 2017; de Munter et al., 2020) and stored at -80ᵒC until use.

**Quantitative RT-PCR (qPCR)**

RNA extraction was performed as previously described from specifically microdissected snap-frozen hippocampus and prefrontal cortex ([Couch et al., 2013](#_ENREF_9), 2016). mRNA was extracted by using TRI Reagent (Molecular Research Center, Inc., Cincinnati, OH, USA). First strand cDNA synthesis was performed using random primers and Superscript III transcriptase (Invitrogen, Darmstadt, Germany); 1 μg total RNA was converted into cDNA. Standard curves were generated using total cDNA to enable normalization to three housekeeping genes glyceraldehyde-3-phosphate (GAPDH), TATA-binding-protein (Tbp), and beta-Actin (ActB) that were selected from four candidates; beta2-microtubulin was excluded to its less stable expression using BioGazelle qBase+ v2.6 software (Biogazelle, Gent, Belgium) and GeNorm algorythm. qPCR was performed using the SYBR Green master mix (Bio-Rad Laboratories, Philadelphia, PA, USA) and the CFX96 Real-time System (Bio-Rad Laboratories, Philadelphia, PA, USA) for IL-1 β, TNF, COX-1, GSK-3 α, GSK-3 β, as well as Mmp9 and Timp1.

Details of primers and cycling conditions can be found in Table 1 (AlcorBio, St.Petersburg, Russia). Data were calculated as relative-fold changes compared to control mice as described elsewhere (Couch et al., 2016, Trofimov et al., 2017). Results of qRT-PCR measurement were expressed as Ct values, where Ct is defined as the threshold cycle of PCR at which amplified product was 0.05% of normalized maximal signal. We used the comparative Ct method and computed the difference between the expression of the gene of interest and the geometric mean of the 3 housekeeping genes in each cDNA sample (2-ΔΔ Ct method). Data are given as expression-folds compared to the mean expression values in control mice. Results are expressed as relative-fold compared to control animals.

***Table 1. Sequences of primers used***

| **Gene** | **Forward primer 5′–3′** | **Reverse primer 5′–3′** |
| --- | --- | --- |
| *GAPDH* | TGCACCACCAACTGCTTAG | GGATGCAGGGATGATGTTC |
| *β-actin* | CTAAGGCCAACCGTGAAAAG | ACCAGAGGCATACAGGGACA |
| *TNF* | GCCTGTAGCCCACGTCGTA | GGCACCACTAGTTGGTTGTCTTTG |
| *COX-1* | GCCTGAGCCCAGATATAGCA | TTTCCGGCTAGAGGTGGGTA |
| *IL-1β* | AACCTGCTGGTGTGTGACGTTTC | CAGCACGAGGCTTTTTTGTTGT |
| *GSK-3β* | TCCATTCCTTTGGAATCTGC | CAATTCAGCCAACACACAGC |
| *GSK-3α* | AATCTTGGCCAGTCTGAGCT | TCAGTCCTGGTGAACTGTCC |
| *Iba-1* | GGCAATGGAGATATCGATA | AGAATCATTCTCAAGATGGC |
| *Mmp9* | CCTCTGCATGAAGACGACAT | GAGGTGCAGTGGGACACATA |
| *Timp1* | CTGGCATAATCTGAGCCCTG | GCAAAGTGATCGCTCTGGTAG |
| *Tbp* | ACCTTCACCAATGACTCCTATG | ATGATGACTGCAATCGC |

The qRT-PCR was performed in a 12 μl reaction volume containing a 10хPCR Buffer (1.2 μl), 25 mmol MgCl2 (2 μl), 10 mmol dNTPs (1 μl), specific forward and reverse primers at 20 pmol/μl concentration (0.5 μl), 10 pmol/μl probe (0.3 μl), cDNA (1 μl), 5 u/μl Taq DNA polymerase (0.5 μl) (Beagle, St. Petersburg, Russia), and ddH2O (5 μl). All samples were run in triplicate. Cycling was performed at 95C° for 5 min followed by a 50-cycle amplification at 95°C for 5 s, then at the annealing temperature 60°C defined previously for 10 s and at the temperature 72°C for 15 s.

**Western Blot**

Tissue samples were treated with lysis buffer containing 20 mM of Tris-HCl (pH 7.5), 450 mM of NaCl, 1%-solution of Triton X-100, 1 mM of EDTA, 1 mM of NaF, 1 mM of Na_3_VO_4,_ and protease inhibitor (Roche Diagnostics, Indianapolis, IN, USA); 50 μl of buffer per 1 g of tissue was used. Samples were then centrifuged at 16 000 rpm for 20 min at 4°C; supernatant was collected and stored until use at -20°C. 25 μg of protein from each sample was mixed with 35 μl of Laemmli buffer. A sample of identical volume, comprising of 26 μl of Laemmli buffer, 5 μl of Page Ruler, and 4 μl of Magic Mark (Sigma, Munich, Germany) was used as a reference. For electrophoresis, samples were diluted in a solution containing MiliQ H_2_O, 1.5 M of Tris Buffer (pH 8.8), 30%-solution of Acrylamide, 10%-solution of SDS Temed, and 10%-solution of ammonium persulfate (APS). For the next step, a solution containing MiliQ H_2_O, 0.5 M of Tris Buffer (pH 8.8), 30%-solution of Acrylamide, 10%-solution of SDS Temed, 10%-solution of APS and gel (Sigma, Munich, Germany) was used. The percentage of gel-containing solution was adjusted to the weight of the protein of interest and was 20% for proteins of the size of 4–40 kDa, 12.5% for proteins of the size of 40–70 kDa, 10% for proteins of the size of 70–100, and 7.5% for proteins over 100 kDa. A buffer containing 25 mM of Tris Base buffer, 192 mM of Glycine (Sigma, Mannheim, Germany), 10%-solution of SDS and MiliQ H_2_O (pH 8.3) was used for gel electrophoresis which was carried out under the constant voltages of 80 V and 130 V.

Polyvinylidene difluoride (PVDF) membrane (9 x 6 cm, EMD Millipore, Billerica, MA, USA) was consequently incubated in a 99%-methanol solution for 1 min (Brocacef, Amsterdam, the Netherlands), a MiliQ H_2_O for 5 min, and a transfer buffer for 15 min. The latter contained 25 mM of Tris Base, 192 mM of glycine, 20%-solution of methanol, and MiliQ H_2_O (pH 8.3). For the next step, blot “transfer sandwich” was composed of buffer-soaked sponge, consisting of two buffer-soaked Whatman filter papers, gel, activated membrane, and ice-cold transfer buffer; a constant current of 300 mA was used for 2 h 30 min.

The membrane was treated with 5%-dry milk solution of TBST, containing 50 mM Tris-HCl (pH=8.2), 150 mM NaCl, 0.05%-solution Tween 20 (Sigma, Munich, Germany) for 1 h at the room temperature and subsequently incubated with primary antibodies (*Table 3*) at 4°C overnight, followed by incubation with respective horseradish peroxidase-conjugated secondary (HRP) antibodies (Sigma-Aldrich, St. Louis, MO, USA) for 2 h at the room temperature on a roller. The membrane was washed in TBST three times, 5 min each time, and then placed on the plastic cover. Then, Western Bright^TM^ ECL kit (Advansta Inc, Menlo Park, CA, USA) was used. Blots were stripped by incubation with Restore Western Blot Stripping Buffer (Thermo Scientific, Rockford, IL, USA) at the room temperature for 15 min. Relative optical density of immunoreactive protein bands was examined using ImageJ software (NIH, Bethesda, MD, USA). Results were normalized to the relative intensity of the β-tubulin band that was selected as a reference protein as described elsewhere (Gorlova et al., 2019).

**Table 2. Primary antibodies used in the Western blot assay**

| **Antibody** | **Dilution** |
| --- | --- |
| Anti-β-tubulin (Abcam, Cambridge, MA, USA) #ab8227 | 1:700 |
| Anti-GSK-3β (Cell Signaling Technology, Beverly, MA, USA) #9832 | 1:800 |
| Anti-GSK-3α (Cell Signaling Technology, Beverly, MA, USA) #4337 | 1:900 |
| Anti-IL-1β (Cell Signaling Technology, Beverly, MA, USA) #12242 | 1:700 |
| Anti-Iba1 (Abcam, Cambridge, MA, USA) ab5076 | 1:500 |

To normalize the data, the relative expression value of each protein of interest was expressed as a percent of the concentration of β-tubulin, which was used as the reference protein. The choice of a reference protein was based on the previous observations where its expression was found to vary moderately across various experimental conditions, as well as the linear representation of the intensity of its signal (Gorlova et al., 2019; Pavlov et al., 2019; de Munter et al., 2020).

**Determination of protein concentration**

Protein concentration was quantified using the BCA protein assay kit (Pierce, Rockford, IL, USA) as described elsewhere (Gorlova et al., 2019; Pavlov et al., 2019, de Munter et al., 2020). The working reagent was prepared in accordance with manufacturer instructions. 25µl of each standard or sample preparations were pipetted into a microplate well; 200µl of the working reagent was added to each well and mixed thoroughly on a plate shaker for 30 seconds; assay was run in duplicates. The covered plate was incubated at 37°C for 30 minutes and cooled to room temperature for 10 min. The absorbance was measured at 562 nm in a Biotek Microplate Reader (Biotek Instruments, Winooski, VT, USA). Ascent Software Program (Winooski, VT, USA) coupled to the microplate reader was used to calculate protein values based comparing optical densities with the standard curve. The standard curve was generated by plotting the average blank-corrected 562 nm measurements for each BSA standard vs. its concentration in µg/ml.

**Administration of drugs**

In the study with FUS-tg mice, potential effects of Neuro-Cells were compared to effects of celecoxib, NSAID and a COX-2 inhibitor, that was used as a classic anti-inflammatory compound (Goldenberg et al., 1999; Pompl et al., 2003), and of riluzole (2-amino-6-trifluoromethoxy-benzothiazole), a standard ALS treatment that can slow down the fatal disease progress by 2-3 months (Miller et al., 2012) and frequently serves as a reference drug in translational studies with ALS (Li et al., 2013; Rodríguez-Cueto et al., 2018). Riluzole tablets (Sandoz, Almere, Netherlands) were crushed and dissolved in tap water, its concentration was adjusted to the dosage of 8 mg/kg/day and daily water intake in CD1 mice (Li et al., 2013; de Munter et al., 2020). Celecoxib-containing food pellets were produced as described elsewhere (Costa-Nunes et al., 2013; Munter et al., 2020), drug concentration was adjusted to the dosage of 30mg/kg/day and daily diet consumption. Intake of solutions and food pellets was measured during the first three days of dosing and then weekly as described elsewhere (Munter et al., 2020).

**Characteristics of human marrow stem cells**

“Neuro-Cells” is a standardized preparation of fresh, unmanipulated human bone marrow-derived stem cells, which was produced under regulations of good manufacturing practices (GMPs) without expansion and/or labeling (de Munter et al., 2019; patent WO2015/059300A1) and provided by Neuroplast BV (Maastricht, Netherlands). Neuro-Cells comprise mesenchymal stem cells and their progenitors (MSCs), hematopoietic stem cells and their progenitors (HSCs), and other mononuclear cells (*Table 3*). The Neuro-Cell preparation comprised 1.39x10^6^ MSCs and HSCs, containing 5x10^5^ CD34^+^ cells in 10 ul. The expression profile of MSC markers is overlapping; in the total cell preparation used in this study, 85.6% were CD105^+^, 13% were CD90^+^, 7% were CD271^+^, and 4% were CD73^+^ in single FACs staining.

**Table 3. Cell populations in ''Neuro-Cells" preparation**

| **Characterization of cell populations in ''Neuro-Cells" preparation** | |
| --- | --- |
| **Cell populations in Neuro-Cells** | **Absolute number of cells injected per mouse in 10**  ul |
| Total number of nucleated cells | 1.39 x 10^6^/10 ul |
|  |  |
| **HSC**: total CD34^+^ cells (calculated by FACs) | 5.00 x 10^5^/10 ul |
| Other mononucleated cells | 8.9 x 10^5^/10 ul |
| The proportion of MSCs expressing CD markers in overlapping manner (in total cell preparation, defined by single staining assay): |  |
| **MSC**: CD271^+^ cells (7%) |  |
| **MSC**: CD90^+^ cells (13%) |  |
| **MSC**: CD105^+^ cells (85.9%) |  |
| **MSC**: CD73^+^ cells (4%) |  |

**Generation of human marrow stem cells**

For production of Neuro-Cells, healthy volunteer donors with informed consent were recruited for collection of 50-75 mL of bone marrow from their iliac crest under local anesthesia following standard operating procedures. Briefly, mononuclear cells were isolated by Ficoll density gradient centrifugation (1.077 g/ml; Sigma, Munich, Germany) at 400×g for 35 minutes. The mononuclear cells were re-suspended in the culture medium composed of DMEM (GIBCO, Rockville, MD, USA) with low glucose concentration and 10% fetal bovine serum (FBS; Hyclone, Logan, UT, USA). The mononuclear cells were plated at 1×10^6^ cells/25 cm^2^ in culture flasks and the cultures were incubated at 37°C in 5% CO_2_ in the air and 95% humidity. The medium was exchanged after 48 hours and then every 3 to 4 days. When the cultures reached approximately 90% of confluence, human marrow stem cells (“Neuro-Cells”) were passaged with 0.25% trypsin (GIBCO) and re-plated into passage culture at a density of 5000 to 10,000 cells/cm^2^. Cells at passages 3 and 4 were used for transplantation. Upon harvest, the cells were isolated by treatment with 0.25% trypsin.

The cells were then washed four times and re-suspended in PBS at a density of 100,000 cells/μl. Viability of the cells was assessed using a 0.4% Trypan Blue dye (Sigma, Berlin, Germany) exclusion method prior to and following transplantation. The cells were analyzed for their immunophenotype by flow cytometry (Beckman Coulter, Fullerton, CA, USA). Fluorescein isothiocyanate (FITC)-conjugated or phycoerythrin-conjugated antibodies specific for human CD14, CD19, CD45, CD34, CD73, CD90, CD105, and HLA-DR tested for flow cytometry application. The percentage of positive cells was determined based on fluorescence emission of the nonspecific FITC/phycoerythrin isotypic antibody controls. A preparation of Neuro-Cells was aliquoted, deeply frozen and stored at -80 C° until the use.

**Intracerebroventricular infusion of Neuro-Cells**

After thawing according to a standard procedure (*see Table 4*), cells were re-suspended in Ringer Lactate buffer and studied for vitality. A counting was performed using the "Countess II FL Automated Cell Counter" (Thermo Fisher Scientific AMQAF1000, Toronto, ON, Canada) according to the manufacturer's protocol. Three batches of Neuro-Cells from the same bone marrow extraction were used in the study. The viability measurement of Neuro-Cells samples used in the experiment has ensured high vitality percentages in samples, that were ranging from 62 to 68%. Subsequent flow cytometry was done and HSC numbers were adjusted in injection suspension prior the infusion of Neuro-Cells to a mouse brain. Therefore, their concentration in buffer was adjusted to 500,000 CD34^+^ in 10 µl for i.c.v. injections; vials were kept in +4C°.

**Table 4: Standard thawing procedure for Neuro-Cells**

| **Nr.** | | **Step 1** | **Set** |
| --- | --- | --- | --- |
| 1 |  |  |  |
|  |  | sterile Ringer Lactate solution is used  pre-warm at 37°C water bath | 50ml  pre-warmed 37°C |

| **Nr.** | | **Step 2** | **Set** |
| --- | --- | --- | --- |
| 1 |  | To thaw frozen Neuro-Cells, cryovials are placed in a water bath at 37°C until only a small sliver of ice is present; caution is paid to prevent cryovial cap from submerging in the water. | 37°C |
| 2 |  |  |  |
|  | A | - Cyovials are transferred to the laminar flow hood  - all vials are wiped with 70% IPA before opening the cap using a 70% IPA soaked tissue |  |
|  | B | each thawed cell type is transferred into an individual 15ml tube containing 5ml pre-warmed thawing buffer | 5ml |
|  | C | - cryovials are rinsed with fresh pre-warmed thawing buffer  - pooled cells are mixed by gently pipetting up/down twice | Rinse 2x |
|  | D | Suspension is centrifuged under 300g for 5 min | 300g 5 min |
|  | E | - supernatant is completely aspirated  - the cell pellet are handled carefully  - pellet is resuspended into the **thawing buffer for washing** |  |
|  | F | For washing, pellet is centrifuged under 300g for 5 min | 300g 5 min |
|  | G | supernatant is completely aspirated  -- pellet is resuspended into the **thawing buffer for washing**  **- cells are pooled where applicable** |  |

Separate preliminary studies were carried out to determine the distribution of infused Neuro-Cells and optimize the protocols for the i.c.v. administration (deMunter et al., 2020). These studies have suggested the presence of Neuro-Cells in the brain and peripheral organs 12 and 24 h after injection of 100, 000 or 250, 000 cells were infused to lateral ventricles. A positive signal in the ventricles, brain tissue, lungs, and spleen was found in Neuro-Cell-treated mice using immunohistochemical assay with human anti-mitochondrial antibodies that was carried out as described elsewhere (Donders et al., 2015). These results suggested a wide distribution of Neuro-Cells in the cerebrospinal fluid and their persistent vitality and were consistent with the results of the pilot study with i.c.v. infusion of bone marrow-derived mouse stem cells obtained from mutants expressing Green Fluorescent Protein (GFP) (de Munter et al., 2020). These experiments also showed that i.c.v. administration of Neuro-Cells to mice at the concentrations 100,000 – 500,000 is well tolerated.

Animals were anesthetized by halothane (Halothane TM; Willy Rusch, Boblingen, Germany) and immobilized in a stereotaxic frame (World Precision Instruments, Sarasota, TX, USA) for unilateral i.c.v. infusion via a hole made in a scull of mice as described elsewhere (Strekalova et al., 2001, 2002). Specifically, after incision of the skin, the periost of the skull was removed and the hole was drilled above the left lateral ventricle of the brain (0.5 mm posterior and 1.0 mm lateral from the midline; coordinates according to Franklin & Paxinos, 1997).

For injection of Neuro-Cells and vehicle, Hamilton syringe (inner diameter: 230.3 µm) was carefully lowered into the hole 1.7 mm below the surface of the skull. Following conditions of Neuro-Cell administration were employed: 10 ul of Neuro-cells suspension (500, 000 cells) in sterile Ringer lactate buffer or buffer alone were infused unilaterally in 10 min. At the end of the injection, the holes were sealed with bone wax and the skin was closed with tissue glue (Histoacryl, B. Braun Surgical, Melsungen, Germany). After surgery, mice were maintained in a warm environment for 3 to 4 h before transferring them back to the animal room.

**SUPPLEMENTARY FIGURES AND TABLES WITH STATISTICAL RESULTS**

**Nissle staining of brains of FUS-rg and wild type control mice**

Nissl staining was performed as described elsewhere (Shelkovnikova et al., 2013). All steps were essentially performed as described previously, five FUS[1-359]-tg 8-weeks-old animals per group were used, paraffin brain blocks were obtained as described elsewhere (Shelkovnikova et al., 2014, 2019). To produce paraffin blocks of dissected brains, PFA-fixed hemispheres were incubated 2 times for 1 h with 70%-ethanol solution, for 1.5 h with 80%-ethanol solution, for 1 h with 95%-ethanol solution, 3 times for 1.5 h, in 100% -ethanol solution, 3 times for 1.5 hour in xylene, for 2 h, in paraffin first wax paraplast Xtra (at 58°C), for 2 h paraffin second wax paraplast Xtra (at 58°C) in cassettes (Sigma-Aldrich, St. Louis, MO, USA). Initially, a small amount of molten paraffin was dispensed in mold from paraffin reservoir. Tissue was transferred into the mold using warm forceps. Subsequently, the mold was placed on a cold plate for paraffin to solidify into a thin layer. Hot paraffin was added to the mold from the paraffin dispenser to cover the front surface of the plastic cassette. Then paraffin blocks were stored until use at the room temperature.

To remove paraffin, sections were incubated 3 times for 5 min with xylene, 2 times for 10 min with 100%-ethanol solution, 2 times for 10 min with 95%-ethanol solution, 2 times for 10 min with 70%-ethanol solution, 2 min for 10 min with 50%-ethanol solution (Sigma-Aldrich, St. Louis, MO, USA) and 2 times for 5 min in ice-cold deionized water. 20-µm-thick hemisphere sections from paraffin brain blocks were obtained from lateral 3.6 to lateral 0.4 mm along the medial-lateral axis ahead of bregma (Paxinos and Franklin, 2001) using a Leica CM 1850 cryostat (Leica Microsystems, Wetzlar, Germany) and mounted on gelatin-covered glass with coverslips. For Nissle assay, brain sections were randomly selected, dewaxed with xylene (Sigma-Aldrich, St. Louis, MO, USA), treated with 95%-ethanol solution (Sigma-Aldrich, St. Louis, MO, USA), stained with 0.1% cresyl violet for 15 min (Sigma-Aldrich, St. Louis, MO, USA) and washed with distilled water. Images were acquired on a Leica SP8 microscope (Leica Microsystems, Mannheim, Germany).

**A B**


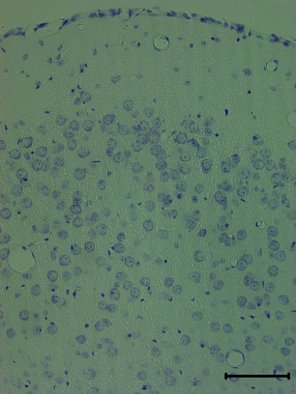

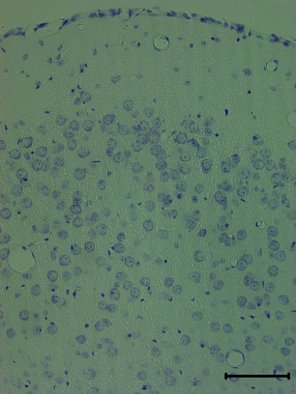


**Supplementary Figure 2.** Nissle staining of frontal lobes of (**A**) the WT mouse and (**B**) FUS[1-359]-tg mouse at age of 8 weeks. No signs of neurodegeneration in FUS[1-359]-tg can be observed; magnification x 200, scale 100 mkm.

**General locomotor activity in the elevated O-maze**

We scored total numbers of crossed sections (4x5.5cm) in both arms of the elevated O-maze during 5 min to estimate possible changes in locomotion between two genotypes. No significant differences were found (df=18, p=0.6091; two-tailed *t*-test; *Suppl. Fig.3*).

**Supplementary Figure 3. A lack of genotype differences in locomotor activity in the O-maze test.** There were no significant differences between groups in the number of crossed sections in O-maze (p>0.05, *t*-test), ten animals per group were used. WT – wild type mice, FUS-tg - FUS[1-359] mice. Bars are Mean ± SEM.

These data show that FUS-tg and wild type control mice had similar response to the testing procedure in the O-maze in terms of locomotion. This lack of changes in general locomotion rules out a possibility of confounds in behavioral analysis of mice in this test.

**Western blot study of protein expression of molecular markers of inflammation and GSK-3 FUS-tg mice treated with old and new anti-inflammatory therapies**

Western blot assay was carried out to investigate the expression of molecular markers of inflammation and GSK-3 in the hippocampus and prefrontal cortex of FUS-tg mice that received celecoxib or Neuro-Cells. To normalize the data, the relative expression value of each protein of interest was expressed as a percent of the concentration of β-tubulin (*see above*) as described elsewhere (de Munter et al., 2020). The choice of a reference protein was based on the previous observations where its expression was found to vary moderately across various experimental conditions, as well as the linear representation of the intensity of its signal (Gorlova et al., 2019; Pavlov et al., 2019; de Munter et al., 2020).

**A Hippocampus**

**B Prefrontal cortex**

**Supplementary Figure 4. Effects of standard ALS treatments and Neuro-Cells on behavioural and brain molecular changes in FUS-tg mice.**

(**A**) Following examination of Western Blot bands in the hippocampus, we found significantly elevated protein levels of IL-1β, Iba-1 and GSK-3β in FUS Veh and FUS Cel animals in comparison with WT Veh mice (p<0.05) that was not found in FUS-tg-NC group (p>0.05). FUS-tg-NC mice had significantly lower expression of these proteins in comparison with other groups of mutants (p<0.05). FUS Cel mice showed significantly higher protein concentration of GSK-3α than mice (p<0.05). (**B**) The analysis of Western Blot bands in the prefrontal cortex has revealed a significant increase of protein levels of IL-1β in GSK-3β in FUS Veh and FUS Cel mice and significant increase of protein concentrations of Iba-1 and GSK-3β in FUS Veh animals in comparison with the WT Veh group (p<0.05). No other group differences were found (p>0.05). β-tubulin was used as a loading control in the Western blot assay, the expression of all proteins is normalized to β-tubulin expression (*p<0.05, vs. WT Veh group, #p<0.05, vs. FUS Veh group: one-way ANOVA and Tukey’s test), see the main ms text; bars are Mean ± SEM.

**Reference list**

Donders R, Vanheusden M, Bogie JF, Ravanidis S, Thewissen K, Stinissen P, Gyselaers W, Hendriks JJ, Hellings N. [Human Wharton's Jelly-Derived Stem Cells Display immunomodulatory Properties and Transiently Improve Rat Experimental Autoimmune Encephalomyelitis.](https://www.ncbi.nlm.nih.gov/pubmed/25310756) Cell Transplant 2015;24:2077-2098.

Couch Y, Anthony DC, Dolgov O, Revischin A, Festoff B, Santos AI, Steinbusch HW, Strekalova T. Microglial activation, increased TNF and SERT expression in the prefrontal cortex define stress-altered behaviour in mice susceptible to anhedonia. Brain Behav. Immun. 2013; 29: 136–146.

Couch Y, Trofimov A, Markova N, Nikolenko V, Steinbusch HW, Chekhonin V, Schroeter C, Lesch KP. Low-dose lipopolysaccharide (LPS) inhibits aggressive and augments depressive behaviours in a chronic mild stress model in mice. J Neuroinflammation. 2016; 13: 1-8.

[Goldenberg MM](https://www.ncbi.nlm.nih.gov/pubmed/?term=Goldenberg%20MM%5BAuthor%5D&cauthor=true&cauthor_uid=10509845). Celecoxib, a selective cyclooxygenase-2 inhibitor for the treatment of rheumatoid arthritis and osteoarthritis. Clin Ther 1999;21:1497-513.

Gorlova A, Pavlov D, Anthony DC, Ponomarev ED, Sambon M, Proshin A, Shafarevich I, Babaevskaya D, Lesсh KP, Bettendorff L, Strekalova T. [Thiamine and benfotiamine counteract ultrasound-induced aggression, normalize AMPA receptor expression and plasticity markers, and reduce oxidative stress in mice.](https://www.ncbi.nlm.nih.gov/pubmed/30817932) Neuropharmacology 2019;156:107543.

[Li J](https://www.ncbi.nlm.nih.gov/pubmed/?term=Li%20J%5BAuthor%5D&cauthor=true&cauthor_uid=23762454), [Sung M](https://www.ncbi.nlm.nih.gov/pubmed/?term=Sung%20M%5BAuthor%5D&cauthor=true&cauthor_uid=23762454), [Rutkove SB](https://www.ncbi.nlm.nih.gov/pubmed/?term=Rutkove%20SB%5BAuthor%5D&cauthor=true&cauthor_uid=23762454). Electrophysiologic biomarkers for assessing disease progression and the effect of riluzole in SOD1 G93A ALS mice. PLoS One 2013;8:e65976.

Malatynska E, Steinbusch H, Redkozubova O, Bolkunov A. Kubatiev A, Yeritsyan NB, Vignisse J, Bachurin S, Strekalova T. [Anhedonic-like traits and lack of affective deficits in 18-month-old C57BL/6 mice: Implications for modeling elderly depression.](http://www.ncbi.nlm.nih.gov/pubmed/22583982) Exp. Gerontol., 2012; 47: 552-564.

Miller RG, Mitchell JD, Moore DH. Riluzole for amyotrophic lateral sclerosis (ALS)/motor neuron disease (MND). Cochrane Database Syst Rev 2012;3:CD001447.

Pavlov D, Bettendorff L, Gorlova A, Olkhovik A, Kalueff AV, Ponomarev ED, Inozemtsev A, Chekhonin V, Lesсh KP, Anthony DC, Strekalova T. [Neuroinflammation and aberrant hippocampal plasticity in a mouse model of emotional stress evoked by exposure to ultrasound of alternating frequencies.](https://www.ncbi.nlm.nih.gov/pubmed/30472146) Prog Neuropsychopharmacol Biol Psychiatry 2019;90:104-116.

Pompl PN, Ho L, Bianchi M, McManus T, Qin W, Pasinetti GM. A therapeutic role for cyclooxygenase-2 inhibitors in a transgenic mouse model of amyotrophic lateral sclerosis. FASEB J 2003;17:725-7.

Paxinos and Franklin, 2001

Strekalova T, Spanagel R, Bartsch D, Henn FA, Gass P. Stress-induced anhedonia in mice is associated with deficits in forced swimming and exploration. Neuropsychopharmacology. 2004; 29: 2007-2017.

Strekalova T, Anthony DC, Dolgov O, Anokhin K, Kubatiev A, Steinbusch HM, Schroeter C. [The differential effects of chronic imipramine or citalopram administration on physiological and behavioral outcomes in naïve mice.](https://www.ncbi.nlm.nih.gov/pubmed/23434605) Behav Brain Res. 2013;245:101-106.

Strekalova T, Bahzenova N. Trofimov A. Schmitt-Böhrer AG, Markova N, Grigoriev V, Zamoyski V, Serkova T, Redkozubova O, Vinogradova D, Umriukhin A, Fisenko V, Lillesaar C, Shevtsova E, Sokolov V, Aksinenko A, Lesch K.-P, Bachurin S. Pro-neurogenic, Memory-Enhancing and Anti-stress Effects of DF302, a Novel Fluorine Gamma-Carboline Derivative with Multi-target Mechanism of Action. Mol Neurobiol. 2018; 55: 335-349.

Veniaminova E, Cespuglio R, Chernukha I, Schmitt-Boehrer AG, Morozov S, Kalueff AV, Kuznetsova O, Anthony DC, Lesch KP, Strekalova T. [Metabolic, Molecular, and Behavioral Effects of Western Diet in Serotonin Transporter-Deficient Mice: Rescue by Heterozygosity?](https://www.ncbi.nlm.nih.gov/pubmed/32132889) Front Neurosci. 2020;14:24.
